# Vibrational Spectroscopic Identification of the [AlCl_2_]^+^ Cation in Ether-Containing Liquid Electrolytes

**DOI:** 10.3390/molecules29225377

**Published:** 2024-11-14

**Authors:** Gabriela P. Gomide, Wagner A. Alves, Andrzej Eilmes

**Affiliations:** 1 Núcleo de Espectroscopia e Estrutura Molecular (NEEM), Departamento de Química, Instituto de Ciências Exatas, Universidade Federal de Juiz de Fora, Juiz de Fora 36036-900, MG, Brazil; 2 Faculty of Chemistry, Jagiellonian University, Gronostajowa 2, 30-387 Kraków, Poland

**Keywords:** vibrational spectroscopy, aluminum chloride, ethereal liquid electrolytes

## Abstract

A Raman and IR study of AlCl_3_-based ethereal solutions is here presented and aims at identifying the [AlCl_2_]^+^ cation, which has been so far unambiguously characterized by ^27^Al NMR spectrometry. To do that, experimental–theoretical vibrational spectroscopy was so employed, and the data are interpreted successfully. As a known amount of water is added to the tetrahydrofuran (THF)-containing electrolyte, a Raman band at 271 cm^−1^ has its intensity increased along with the most intense band of [AlCl_4_]^−^, and such behavior is also seen for a band at 405 cm^−1^ in the IR spectra. New bands at around 420 and 400 cm^−1^ are observed in both Raman and IR spectra for the tetraglyme (G4)-based systems. The [AlCl_2_(THF)_4_]^+^ complex, in the *cis* and *trans* forms, is present in the cyclic ether, while the *cis*-[AlCl_2_(G4)]^+^ isomer is identified in the acyclic one.

## 1. Introduction

Ethereal aluminum chloride solutions have been used in Al electrodeposition baths since the investigation performed by Couch and Brenner in 1952 [1]. Most recently (2014), the addition of magnesium chloride to those systems allowed researchers to easily synthesize the first Mg battery electrolyte with all-inorganic salts, termed the magnesium aluminum chloride complex (MACC) [2]. Various Mg(II) complexes and [AlCl_4_]^−^ have been identified in this electrolyte [3,4,5,6,7], but the MgCl_2_:AlCl_3_ ratio must be very well tuned in order to avoid the presence of [AlCl_2_]^+^, which seems to be responsible for corrosion of the Mg anode [8]. Not surprisingly therefore, such a cation and [AlCl]^2+^ have been proposed in AlCl_3_-based ethereal systems in accordance with the reactions (1) and (2):

2AlCl_3_ ⇋ [AlCl_2_]^+^ + [AlCl_4_]^−^ (1)

3AlCl_3_ ⇋ [AlCl]^2+^ + 2[AlCl_4_]^−^ (2)

^27^Al NMR spectrometry has been widely used in the study of aqueous and nonaqueous solutions of aluminum salts. For instance, Nöth and coworkers [9] investigated AlCl_3_/tetrahydrofuran (THF) solutions and three distinct peaks were identified: the most intense one at 64 ppm was related to pentacoordinated Al (AlCl_3_·2THF), whereas a sharp line at 103 pm resulted from the presence of a tetracoordinated species ([AlCl_4_]^−^); a rather broad signal at around 19 ppm was interpreted on the basis of a hexacoordinated Al environment, which could be either [AlCl_2_(THF)_4_]^+^ or [AlCl(THF)_5_]^2+^. Replacing the solvent for mono-, di- and triglyme, only the sharp peak at 103 and a broad one at 25 ppm were detected. Those authors concluded that AlCl_3_ dissociation into [AlCl_4_]^−^ and solvated [AlCl_2_]^+^ and/or [AlCl]^2+^ increases as a function of the ether chelating effect. As expected, the ^27^Al NMR spectrum reported by Bieker and colleagues [8] for an AlCl_3_/tetraglyme (G4) solution showed the two same signals, but the corresponding Raman spectrum revealed only the presence of the tetrahedral anion. Similar vibrational spectroscopic results were reached by Kitada et al. [10], who also investigated the electrochemical properties of AlCl_3_ solutions in selected glymes. However, the structures of the [AlCl_2_(glyme)_n_]^+^ complexes were based on the ^27^Al NMR data reported by other researchers [9].

So far, one can notice that [AlCl_4_]^−^ has been easily identified in the Raman and IR spectra, in contrast to the cationic species. Derouault and Forel [11] synthesized the solid AlCl_3_·THF and AlCl_3_·2THF compounds, and the vibrational analysis suggested that the former has a molecular structure, whereas the latter may be described as an [AlCl_2_(THF)_4_^+^, AlCl_4_^−^]-type ionic arrangement. At the same year, Derouault and coauthors [12] spectroscopically investigated AlCl_3_ solutions in THF and in THF–dichloromethane mixtures. Owing to the use of a visible radiation laser, the Raman spectra were strongly disturbed by fluorescence, and so AlCl_3_·2THF and [AlCl_4_]^−^ were the only species identified. On the other hand, weak signals near 440 and 360 cm^−1^ were observed in the IR spectra and related to the [AlCl_2_(THF)_4_]^+^ cation. Curiously, the absorption spectra are shown up to 300 cm^−1^, even though those authors have also employed polyethylene windows.

By using a near-IR radiation laser, Alves et al. [13] have recorded fluorescence-free Raman spectra of AlCl_3_ solutions in THF. Despite this advantage, the spectra were also characterized by the neutral and anionic species. Hence, we decide in this work to acquire Raman spectra with laser power equal to 1W, which is twice larger than the one previously used, aiming at identifying the cationic species in ethereal solvents with different coordinating abilities. The IR technique is also combined to Raman data in order to discuss aspects related to molecular isomerism. All experiments are supplemented by quantum chemical (QC) calculations employing density functional theory (DFT) and ab initio molecular dynamics (AIMD) simulations, and it is expected that information drawn from this study may be useful for the advance in the development of electrolytes employed in rechargeable Al(III) and Mg(II) batteries.

## 2. Results

### 2.1. Experimental Spectra

#### 2.1.1. AlCl_3_-THF System

**Figure 1:**
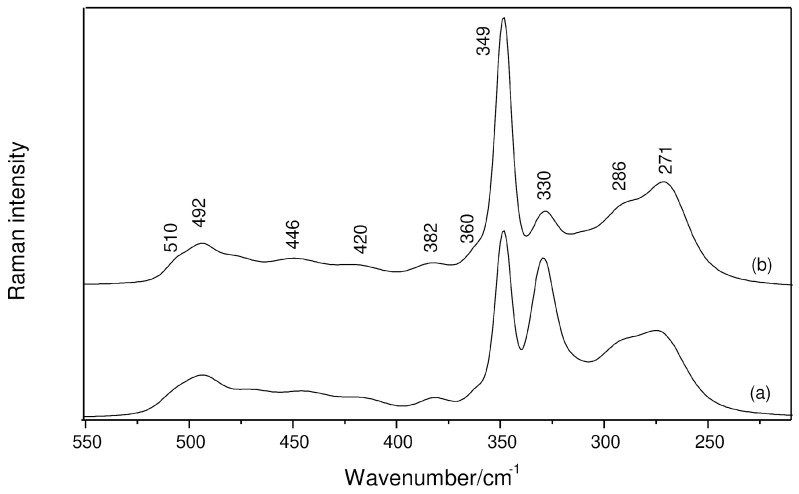
Raman spectra of a 0.5 mol kg^−1^ AlCl_3_ solution at the region of the ν_AlO_ and ν_AlCl_ vibrations: (a) AlCl_3_-THF system; (b) AlCl_3_-THF:H_2_O system with water/salt molar ratio of 0.4.

The interaction of THF with AlCl_3_ is typically evidenced in the Raman spectrum by the bands at 858, 927 and 1042 cm^−1^, which arise from the perturbation provoked by the solute to the ν_CO_ + ν_CC_ stretching vibrations of the ether. These features have been observed in the present work, but considering that they were previously discussed in detail [13] and such wavenumbers had already been reported by other authors [12], the spectral range 1100–800 cm^−1^ will not be shown. On the other hand, the region containing the ν_AlO_ and ν_AlCl_ modes is illustrated in Figure 1, where very weak signals between 510 and 360 cm^−1^ are seen along with intense bands going from 349 to 271 cm^−1^. It is important to stress that many of these components were not earlier identified by using a laser power of 0.5 W [13]. For instance, the weakest Raman signal of [AlCl_4_]^−^ at 492 cm^−1^, which is assigned to the asymmetric ClAlCl stretching mode, ν_as(ClAlCl)_, can be now observed in the spectra. The symmetric vibration (ν_s(ClAlCl)_) located at 349 cm^−1^, which was previously observed and is the most intense band in this region, is accompanied by another appreciable intensity band at 330 cm^−1^ (Figure 1(a)), besides others at 286 and 271 cm^−1^. The former band is attributed to the ν_s(ClAlCl)_ mode of AlCl_3_·THF and AlCl_3_·2THF [11,12,14,15], whereas the second belongs to the solvent (Figure S1). The third is reported for the first time in the literature and may be due to the presence of a cationic complex. Regarding the fact that charged species are favored by dilution [13], a known amount of water was further added to the AlCl_3_-THF system (details are mentioned in Materials and Methods). As can be seen in Figure 1(b), the 330 cm^−1^ band has its intensity significantly reduced, whereas the bands at 349 and 271 cm^−1^ are enhanced. Furthermore, the band intensity ratio (*I*_271_/*I*_349_ = 0.4) is the same in the absence and presence of water, thus suggesting that only one of the equilibria above (reactions (1) and (2)) takes place. A moderate-intensity Raman band at 284 cm^−1^ was assigned by Derouault and Forel [11] to the ν_s(ClAlCl)_ mode of solid AlCl_3_·2THF in an [AlCl_2_(THF)_4_^+^, AlCl_4_^−^]-type ionic arrangement. Since bond distances are overall longer in solution, the 271 cm^−1^ band could be then related to the monovalent cationic Al(III) complex.

**Figure 2:**
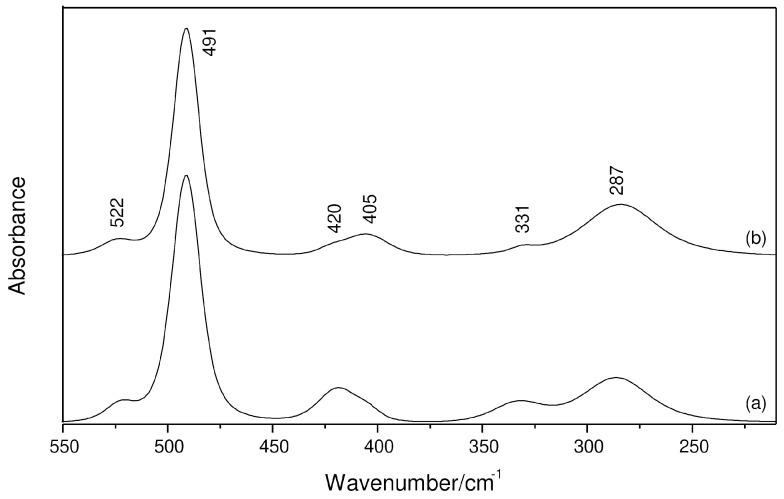
Far-IR spectra of a 0.5 mol kg^−1^ AlCl_3_ solution at the region of the ν_AlO_ and ν_AlCl_ modes: (a) AlCl_3_-THF system; (b) AlCl_3_-THF:H_2_O system with water/salt molar ratio equal to 0.4.

Far-IR spectra of the same solutions were also obtained and are shown in Figure 2. As expected, the 491 cm^−1^ band of [AlCl_4_]^−^ is now the most intense one in the considered spectral range, whereas the 349 cm^−1^ signal is IR-inactive. The former comes along with a satellite at 522 cm^−1^, which is attributed to the ν_AlO_ mode [16,17] of both neutral and cationic complexes. However, the band related to THF at 287 cm^−1^ (Figure S1) is single, and the 271 cm^−1^ signal cannot be identified in the absence (Figure 2(a)) and presence (Figure 2(b)) of water, strongly indicating the abundance of a *trans*-[AlCl_2_(THF)_4_]^+^ complex in the media. The intensity of the band at 331 cm^−1^ is again decreased as water is added to the system, and this behavior is also observed for a band at 420 cm^−1^. In contrast, the 405 cm^−1^ band, which appears as a shoulder in the water-free system, becomes more intense than the component on the higher wavenumber side.

#### 2.1.2. AlCl_3_-G4 System

**Figure 3:**
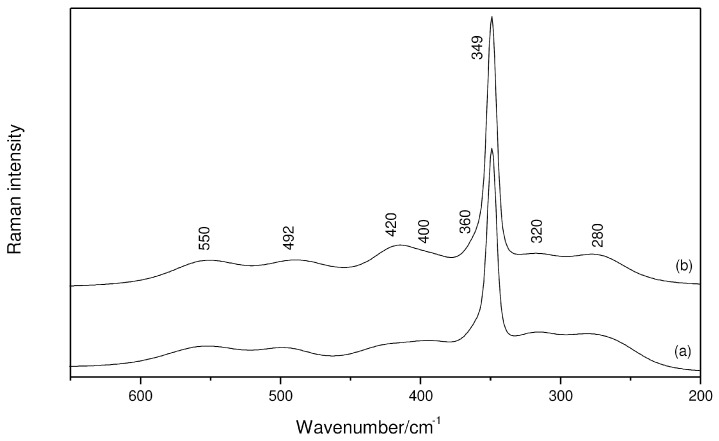
Raman spectra of AlCl_3_/G4 solutions at the region of the ν_AlO_ and ν_AlCl_ vibrations: (a) 1 mol kg^−1^; (b) 2 mol kg^−1^.

The perturbation of AlCl_3_ to the coordination sites of G4 (CO oscillator) is vibrationally characterized by Raman bands at 840 and 870 cm^−1^, which have already been reported by Kitada and colleagues [10]. Here, special attention is paid to Raman bands at 420, 400 and 320 cm^−1^, which also behave in different ways when G4 is employed as a solvent (Figure 3). That is, the first pair of bands has the increased intensity along with the most intense band of [AlCl_4_]^−^, but the latter signal is smoothly decreased with increasing salt molality. Such a trend indicates the presence of an equilibrium between cationic and neutral complexes, where the wavenumber observed for the non-charged species is now downshifted by 10 cm^−1^ due to the chelating effect of G4, which weakens the AlCl oscillator. The use of this solvent leads to the appearance of the bands at 550 and 280 cm^−1^ (Figure S2) and, as can be seen, the lower wavenumber component would make hard the identification of the band observed at 271 cm^−1^ ([AlCl_2_]^+^) in the presence of THF.

**Figure 4:**
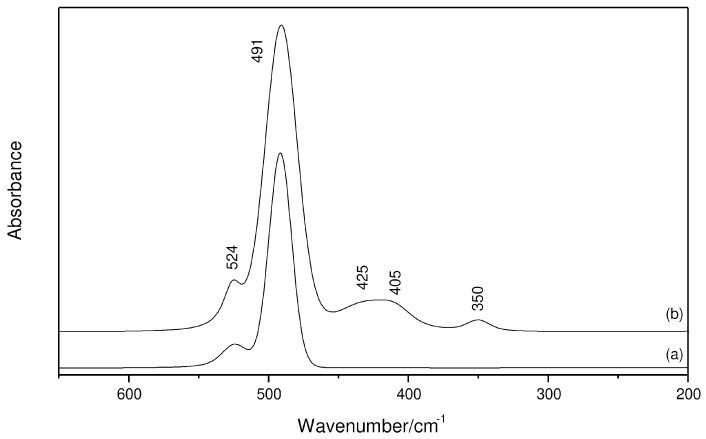
Far-IR spectra of AlCl_3_/G4 solutions at the region of the ν_AlO_ and ν_AlCl_ vibrations: (a) 1 mol kg^−1^; (b) 2 mol kg^−1^.

Evidence for the chemical bond formation between the aluminum halide and G4 is here given by the ν_AlO_ vibration, which arises in the IR spectra as a band at 524 cm^−1^ (Figure 4). It is accompanied by a single signal at 491 cm^−1^ ([AlCl_4_]^−^) at the 1 mol kg^−1^ composition (Figure 4a), but three new bands at 425, 405 and 350 cm^−1^ compose the spectrum of the 2 mol kg^−1^ AlCl_3_/G4 solution (Figure 4b). One clearly observes a good relationship between the first two bands and those identified at 420 and 400 cm^−1^ in the Raman spectra, and the third feature must be overlapped by the strong signal of the anion (Figure 3). As also evidenced by the ^27^Al NMR spectra [8,10], the neutral Al(III) species is not observed in the IR spectra, thus confirming that charged complexes are the most abundant in glymes and ruling out any need to add water to this system.

### 2.2. Simulated Spectra

**Figure 5:**
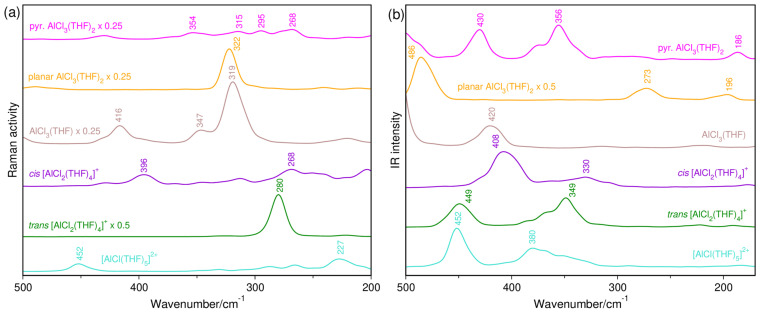
AIMD-simulated Raman (**a**) and IR (**b**) spectra of AlCl_n_ complexes with explicit THF solvent molecules.

From AIMD simulations (details in Materials and Methods), Raman and IR spectra of AlCl_n_ complexes in THF were calculated and are shown in the Figure 5a,b, respectively, and their optimized structures are illustrated in Figure S3. Strong Raman activity at ~320 cm^−1^ is predicted for the AlCl_3_(THF) and planar AlCl_3_(THF)_2_ complexes, whereas several weaker bands are obtained for the pyramidal AlCl_3_(THF)_2_ structure (“planar” and “pyramidal” refer to the AlCl_3_ core in the complexes). A sole peak at 420 cm^−1^ in the IR spectrum of AlCl_3_(THF) may be correlated to a weaker Raman band calculated at 416 cm^−1^. The binding of the second THF molecule leads to the arising of two moderate signals at 273 cm^−1^ and 356 cm^−1^ in the IR spectra of planar and pyramidal AlCl_3_(THF)_2_, respectively. The formation of the former complex is slightly disfavored (ΔG*_f_* = 0.5 kcal/mol for THF binding, i.e., at the limit of the accuracy of calculations), whereas the cost for the formation of the latter is higher and reaches 4 kcal/mol. The [AlCl(THF)_5_]^2+^ complex exhibits a peak at 452 cm^−1^, which is weak in the Raman spectrum and strong in the IR one; a weaker IR band positioned at 380 cm^−1^ can be still identified. An intense Raman peak at 280 cm^−1^ was calculated for the *trans*-[AlCl_2_(THF)_4_]^+^ complex, and due to its nearly centrosymmetric structure, no IR intensity is obtained in this wavenumber, so that only bands at ~350 and 450 cm^−1^ appear in the spectrum. On the other hand, the Raman activity of *cis*-[AlCl_2_(THF)_4_]^+^ is spread over some rather weak bands, and its strongest IR signal is calculated at 408 cm^−1^. Although its free energy is about 8 kcal/mol higher than that of the *trans* aggregate, their relative stabilities show dependence on the presence of the counterion. The free energy difference between the *cis* and *trans* [AlCl_2_(THF)_4_][AlCl_4_] aggregates drops to 2.7 kcal/mol, and the potential energy of the *cis* form is now 0.2 kcal/mol lower than the one of the *trans* structures. The effect can be attributed to the better electrostatic stabilization of the *cis* complex, in which, unlike the *trans* aggregate, the [AlCl_4_]^−^ anion can be located away from the negatively charged Cl atoms of the AlCl_2_ moiety (Figure S4). QC-calculated vibrational spectra for both isomers with and without the counterion exhibit very similar patterns, except for the presence of the anion bands (Figure S5). So, analogous behavior is also expected for the AIMD-simulated spectra.

**Figure 6:**
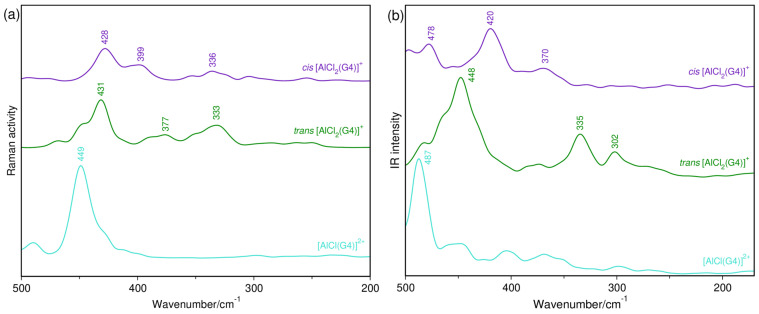
AIMD-simulated Raman (**a**) and IR (**b**) spectra of AlCl_n_ complexes with explicit G4 solvent molecule.

Vibrational spectra simulated for ion aggregates in G4 are shown in Figure 6, and their corresponding structures are available in Figure S6. Intense bands at ~450 (Raman spectrum) and ~490 cm^−1^ (IR spectrum) characterize the [AlCl(G4)]^2+^ species. The Raman spectra of [AlCl_2_(G4)]^+^, in the *cis* and *trans* forms, show a major band at about 430 cm^−1^, but a shoulder at ~400 cm^−1^ can be seen for the former. Additional IR information reveals that a very strong signal at around 450 cm^−1^ is characteristic of the second isomer. The charged *trans* structure is only 0.5 kcal/mol lower than the *cis* form, so that the free energy difference at the level of calculations is almost negligible. With the presence of the [AlCl_4_]^−^ anion, the free energy of the *cis* form becomes 3.7 kcal/mol lower than that of the *trans* complex, indicating the better stability of the former structure.

**Figure 7:**
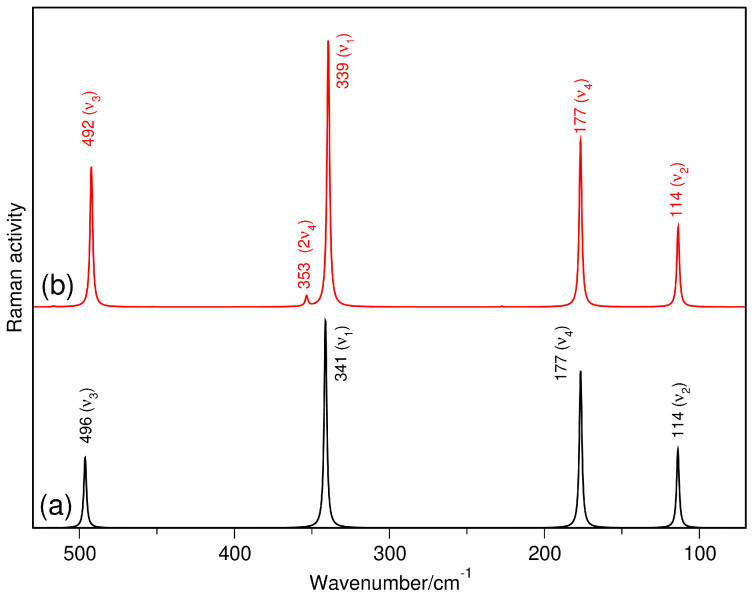
Calculated harmonic (a) and anharmonic (b) Raman spectrum of the [AlCl_4_]^−^ anion.

During this investigation the behavior of the experimental Raman band at 360 cm^−1^, which is seen in both ethers, has attracted our attention. That is, its inactivity in the IR spectra comes along with the most intense band of [AlCl_4_]^−^ at 349 cm^−1^. Furthermore, it is insensitive to the solvent change and this fact strongly suggests that no ether molecule is coordinated to Al(III). In order to elucidate such spectral behavior, harmonic and anharmonic Raman spectra were calculated for the tetrahedral anion (Figure 7). The four bands predicted by symmetry are present in both spectra, but only one intense signal at 341 cm^−1^ is observed in the harmonic model for the ν_1_ region (Figure 7a). Unlike, the highest signal of this anion at 339 cm^−1^ is accompanied by a very weak peak at 353 cm^−1^ in the anharmonic spectrum (Figure 7b). The latter model is in excellent agreement with the experimental result and allows us to guarantee that the 360 cm^−1^ band corresponds to the overtone (2ν_4_) of the experimental band located at 180 cm^−1^.

## 3. Discussion

The extremely low Raman-scattering cross sections and the IR bands simulated at 430 and 356 cm^−1^ indicate that the formation of the pyramidal AlCl_3_(THF)_2_ complex is unlikely, and such findings are also corroborated by its value of ΔG*_f_*. Actually, the experimental and calculated spectra reveal that AlCl_3_(THF) is the major neutral complex in solution, but tiny amounts of planar AlCl_3_(THF)_2_ may be still present. In other words, the theoretical Raman peak at around 320 cm^−1^, which is the most intense feature of both complexes, is in line with the experimental Raman band observed at 330 cm^−1^, which is also IR-active, and its intensity is strongly dependent on the dilution. Similar dependence can also be seen for a 420 cm^−1^ band, which is simulated at 416 and 420 cm^−1^ in the respective Raman and IR spectra of the tetrahedral neutral complex. Since no peak has been calculated at these positions for the bipyramidal neutral compound, we believe that it is either absent or its concentration is too low. If the latter condition is true, its most intense IR peak at 486 cm^−1^ will be overlapped by the 491 cm^−1^ band of the [AlCl_4_]^−^ anion. Despite our argument, Derouault et al. [12] have reported very strong and medium IR peaks at 490 and 420 cm^−1^, respectively, for a *trans*-AlCl_3_·2THF isomer.

The 271 cm^−1^ band is so related to the [AlCl_2_(THF)_4_]^+^ species not only because it is enhanced by dilution, but also due to its good relationship with the band simulated at 280 cm^−1^ for the *trans* isomer. Its high intensity in the Raman spectrum as well as inactivity in the IR one is expected for a totally symmetric vibration. At the same time, the 405 cm^−1^ band, which is also dependent on the dilution, is IR-active and Raman-inactive, and so it could be assigned to the asymmetric vibration of such an isomer. Nevertheless, it shows good correlation with a peak calculated at 408 cm^−1^, which actually belongs to the *cis* isomer. Taking into account that the free energy difference in the isomers in the presence of [AlCl_4_]^−^ suggests the coexistence of both *trans* and *cis* forms in solution, their Raman-scattering and IR-absorption cross sections seem to be then determining for this case. Indeed, the 280 cm^−1^ band reveals that the *trans* isomer is a better scatterer than the *cis* form, but the latter is a greater absorber, as pointed out by the 408 cm^−1^ band. Similar trends are also observed from QC-calculated IR and Raman spectra (Figure S5).

The majority presence of both anionic and cationic complexes in G4-based systems is here evidenced by vibrational spectroscopy for the first time. The asymmetric IR band measured at 425 cm^−1^ shows good relationship with the asymmetric IR peak simulated at 420 cm^−1^, whereas the Raman bands observed at 420 and 400 cm^−1^ are very well described by the Raman signals calculated at 428 and 399 cm^−1^ for *cis*-[AlCl_2_(G4)]^+^. These results combined with the ΔG*_f_* value of this isomer, in the presence of the counterion, strongly suggest its abundance in the investigated solutions.

Comparison between the calculated and experimental data leads to the conclusion that the spectral features of [AlCl]^2+^ are not observed in either solvent. Therefore, an appreciable abundance of the doubly charged complex is rather unlikely, thus minimizing the practical importance of the equilibrium (2) in ether-containing electrolytes. Although the chelating effect of the glyme stabilizes the ionic species, the low dielectric constant values of THF and G4, which are very close to each other (see Computational Details), are not capable of providing higher oxidation state species. Our statement is supported by a study of AlCl_3_/acetonitrile solutions [18], in which the [AlCl(CH_3_CN)_5_]^2+^ and [Al(CH_3_CN)_5-6_]^3+^ complexes are proposed from vibrational and ^27^Al and ^35^Cl NMR spectroscopies. In spite of the higher dielectric constant of the nitrile (36.7), reliable conclusions were not reached due to the large vibrational band overlapping and difficulties in recording accurate NMR data.

The anharmonic Raman spectrum calculated for [AlCl_4_]^−^ was very useful in the assignment of the 360 cm^−1^ band (2ν_4_), which is usually identified as a shoulder on the higher wavenumber side of the most intense band (ν_1_) of this anion. Here, it is important to mention its presence in the Raman spectra of MACC-based electrolytes [3,5,6,8], which has been assigned to the ν_MgO_ vibration of soluble Mg(II) complexes [16,17]. However, our investigation clearly shows that both ν_MgO_ and 2ν_4_ modes compose the shoulder observed in the Raman spectra of those electrolytic solutions.

All the bands discussed in this work are summarized in Table 1 and Table 2 for convenience.

## 4. Materials and Methods

### 4.1. Experimental Details

Tetrahydrofuran (THF, ≥99.9%), tetraethylene glycol dimethyl ether (tetraglyme (G4), ≥99%) and aluminum trichloride (AlCl_3_, 99.99%) were purchased from Sigma-Aldrich, St. Louis, MO, USA. Salt compositions are expressed as molalities in order to better compare with systems already studied. As pointed out from the composition-dependent Raman spectra, the dissociation degree at a 0.5 mol·kg^−1^ AlCl_3_/THF solution is substantially high [13]. Hence, such a composition was chosen for analysis, followed by the addition of water in order to obtain a maximum water/salt molar ratio equal to 0.4. Larger quantities of the protic solvent make the solution turbid due to the formation of aluminum hydroxides. No water was delivered to AlCl_3_/G4 solutions because the glyme stabilizes the ionic species in a certain degree [8,9,10].

Far-IR spectra were recorded on a Bruker FTIR Vertex 80v spectrometer using polyethylene windows. Raman spectra were acquired on a Bruker FT-Raman MultiRAM using the 1064 nm line of the Nd:YAG laser with power equal to 1 W. A Ge detector operating at liquid nitrogen temperature was also employed, and the solutions were inserted into sealed NMR tubes. Both far-IR and Raman spectra were obtained with 2 cm^−1^ resolution at the temperature of 25 ± 2 °C.

### 4.2. Computational Details

Quantum chemical computations using the Gaussian 09 rev. D01 program [19] were employed to obtain optimized geometries of solvated neutral or ion aggregates and vibrational frequencies. DFT methodology with ωB97XD functional and aug-cc-pVDZ basis set was applied. Complexes of Al(III) cations with Cl anions were explicitly solvated with a few (1–5) solvent (THF or G4) molecules. To account for the bulk solvent, all structures were embedded in an implicit solvent modeled via the Polarizable Continuum Model (PCM) approach. Values of 7.43 and 7.80 were used for static dielectric constant of THF and G4, respectively. Gibbs free energies of solvated aggregates were calculated based on the energies and vibrational frequencies of the optimized structures.

Additional QC calculations of harmonic and anharmonic vibrational frequencies were performed for [AlCl_4_]^−^ anion in vacuum at the ωB97XD/6-31+G* level of theory using Gaussian 16 rev. A.03 [20].

Ab initio molecular dynamics simulations were performed for selected solvated ion complexes. Terachem v. 1.93 [21] running on NVIDIA Tesla K40d GPUs was used with ωB97X functional and the 6-31+G* basis set. Implicit solvent was modeled via the COSMO approach using the same values of dielectric constant as in QC calculations. All systems were initially equilibrated in 10 ps simulations with a time step of 1 fs at T = 298 K using the Langevin thermostat. Then, MD simulations in the NE ensemble continued for another 20 ps with a time step of 0.5 fs. IR and Raman spectra were computed as Fourier transforms of the autocorrelation function of the total dipole moment and the polarizability tensor, respectively, calculated for every second frame of the NE trajectory (i.e., with a time step of 1 fs).

## 5. Conclusions

The use of an FT-Raman instrument with maximum laser power (1W) allowed us to characterize the bands of the [AlCl_2_]^+^ cation in AlCl_3_ -containing ethereal solutions. By combining such results with far-IR data and theoretical investigations by QC-calculations and AIMD simulations, it was possible to obtain a conclusion on the main species in the media, and a good relationship with information obtained by ^27^Al NMR spectrometry was reached. In THF, appreciable amounts of AlCl_3_(THF), besides [AlCl_4_]^−^ and the monovalent cationic complex, in the *cis* and *trans* forms, were identified, whereas the *cis* isomer and the anion were the most abundant in G4. The absence of the [AlCl]^2+^ cation was interpreted on the basis of the low dielectric constant values of the selected ethers, but further investigations in the presence of higher dielectric constant additives may lead to the formation of this species. Indeed, such a strategy has been used to improve the electrochemical performance of rechargeable Mg battery electrolytes [22] and is also in our plans. Finally, regarding the fact that the Raman technique has been very exploited in the materials science area, the band at 271 cm^−1^ may be then used as a marker to monitor the presence of [AlCl_2_]^+^, which is deemed a deleterious species for the Mg anode.

## Figures and Tables

**Table 1:** Wavenumbers (in cm^−1^) of vibrational bands measured and calculated for species present in the AlCl_3_-THF and AlCl_3_-THF:H_2_O systems.

Experimental		Calculated		Chemical Species
Raman	IR	Raman	IR	
271		280		*Trans*-[AlCl_2_(THF)_4_]^+^
286	287			THF solvent
330	331	319 322		AlCl_3_(THF) *Planar*-AlCl_3_(THF)_2_
349		339		[AlCl_4_]^−^
360		353		[AlCl_4_]^−^
382				N.A.^1^
	405		408	*Cis*-[AlCl_2_(THF)_4_]^+^
420	420	416	420	AlCl_3_(THF)
446				N.A.^1^
492	491	492	492	[AlCl_4_]^−^
510				N.A.^1^
	522			AlCl_3_(THF) *Planar*-AlCl_3_(THF)_2_ *Cis*-[AlCl_2_(THF)_4_]^+^

^1^ N.A. = Not assigned.

**Table 2:** Wavenumbers (in cm^−1^) of vibrational bands measured and calculated for species present in AlCl_3_/G4 solutions.

Experimental		Calculated		Chemical Species
Raman	IR	Raman	IR	
280				G4 solvent
320				AlCl_3_(G4)
349		339		[AlCl_4_]^−^
	350		370	*Cis*-[AlCl_2_(G4)]^+^
360		353		[AlCl_4_]^−^
400	405	399		*Cis*-[AlCl_2_(G4)]^+^
420	425	428	420	*Cis*-[AlCl_2_(G4)]^+^
492	491	492	492	[AlCl_4_]^−^
	524			*Cis*-[AlCl_2_(G4)]^+^
550				G4 solvent

## Data Availability

The raw data supporting the conclusions of this article will be made available by the authors on request.

## Supplementary Materials

The following supporting information can be downloaded at: https://www.mdpi.com/article/10.3390/molecules29225377/s1, Figure S1: Raman and IR spectra of liquid THF in the spectral range 550–210 cm^−1^; Figure S2: Raman and IR spectra of liquid G4 in the spectral range 650–200 cm^−1^; Figure S3: Structures of AlCl_n_ solvates in THF obtained from the QC calculations at the ωB97XD/aug-cc-pVDZ level; Figure S4: Structures of neutral [AlCl_2_]^+^ solvates in THF and in G4 obtained from the QC calculations at the ωB97XD/aug-cc-pVDZ level; Figure S5: IR and Raman spectra of charged and neutral [AlCl_2_]^+^ solvates in THF and in G4 obtained from the QC calculations at the ωB97XD/aug-cc-pVDZ level; Figure S6: Structures of AlCl_n_ solvates in G4 obtained from the QC calculations at the ωB97XD/aug-cc-pVDZ level.
