# Supplementary material for: Vibrational Spectroscopic Identification of the [AlCl2]+ Cation in Ether-Containing Liquid Electrolytes
Source: Molecules. 2024 Nov 14;29(22):5377. doi: 10.3390/molecules29225377 (PMC11597026; doi:10.3390/molecules29225377)
Supplement: Supplementary file 1 [file molecules-29-05377-s001.zip › molecules-3302068-supplementary.pdf]

Vibrational spectroscopic identification of the $[AlCl_2]^+$ cation in ether-containing liquid electrolytes

Gabriela P. Gomide, Wagner A. Alves and Andrzej Eilmes

Supplementary Material

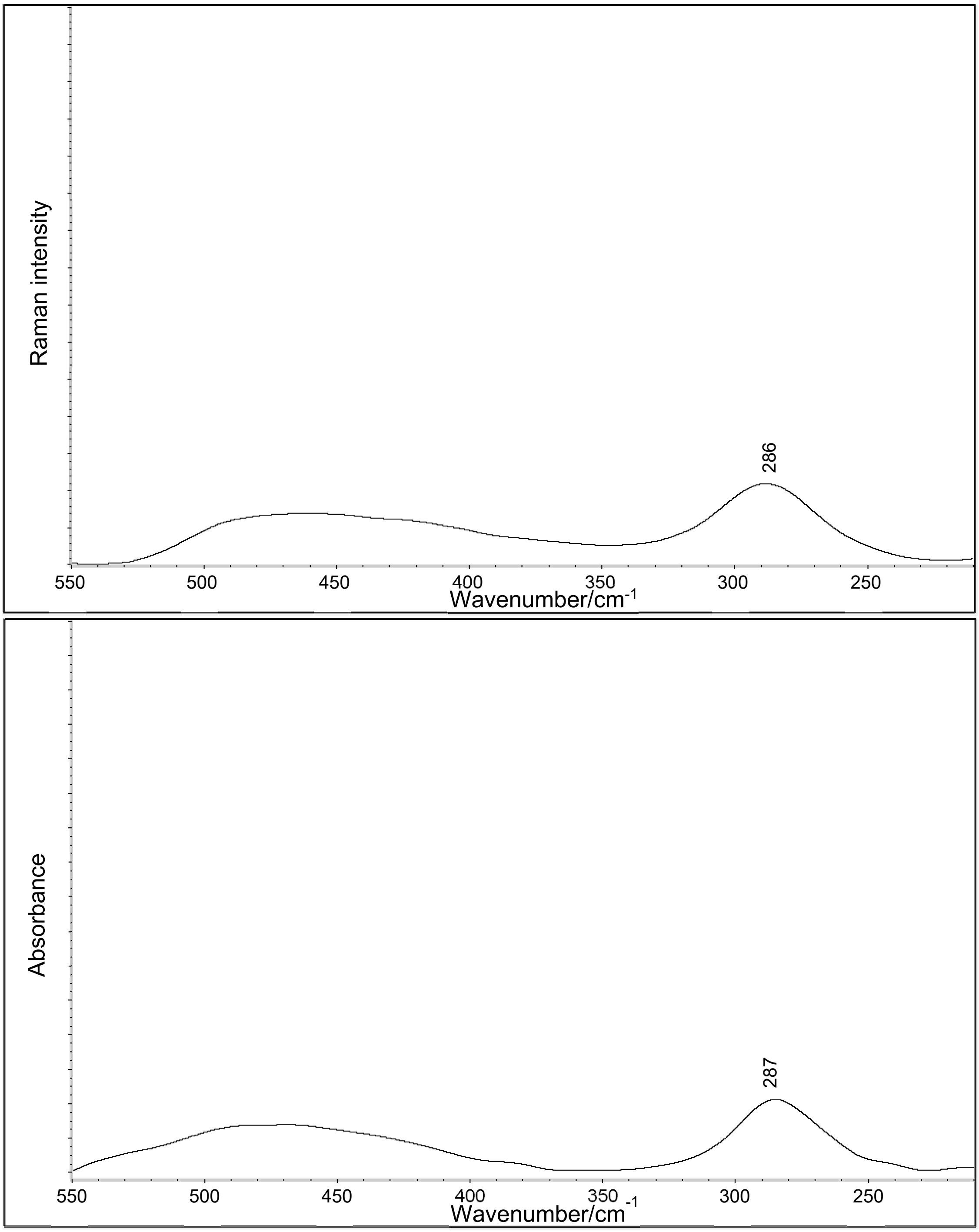

Figure S1. Raman (top) and IR (bottom) spectra of liquid THF in the spectral range 550-210 cm^{-1}.

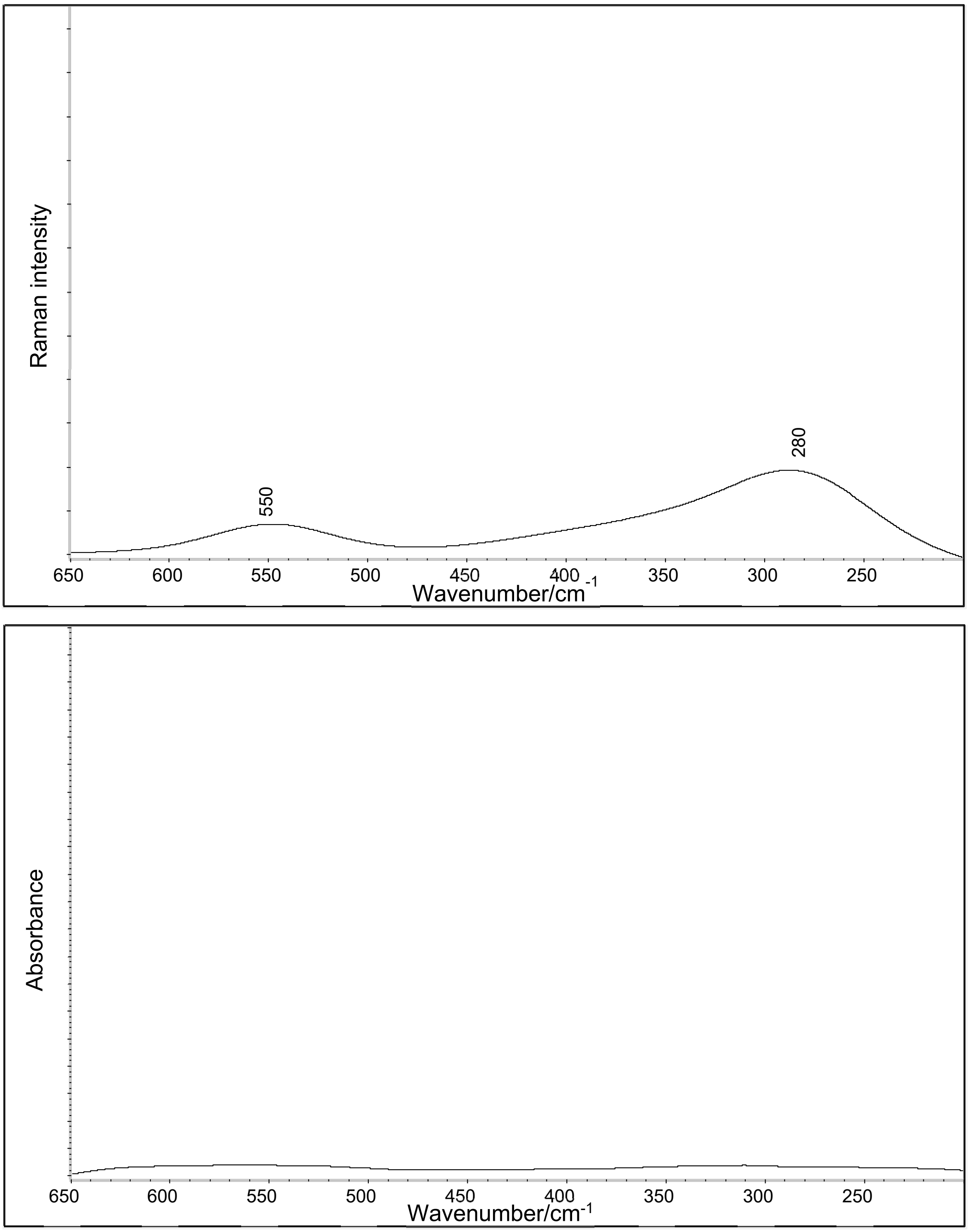

Figure S2. Raman (top) and IR (bottom) spectra of liquid G4 in the spectral range 650-200 cm^{-1}.

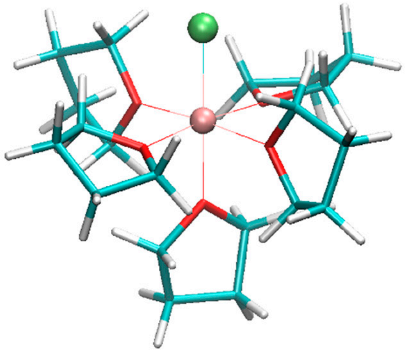

$[AlCl(THF)_5]^{2+}$

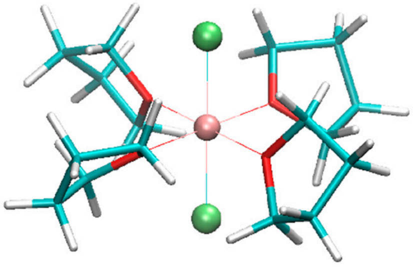

trans-$[AlCl_2(THF)_4]^+$

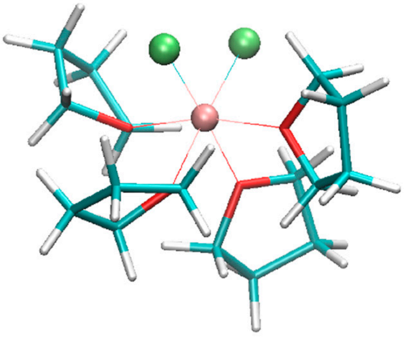

cis-$[AlCl_2(THF)_4]^+$

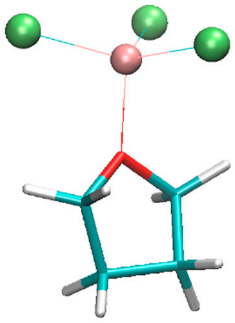

$AlCl_3(THF)$

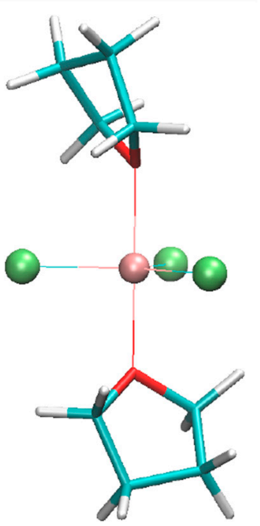

planar $AlCl_3(THF)_2$

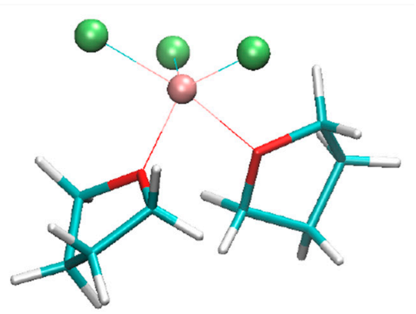

pyramidal $AlCl_3(THF)_2$

Figure S3. Structures of $AlCl_n$ solvates in THF obtained from the QC calculations at the ωB97XD/aug-cc-pVDZ level.

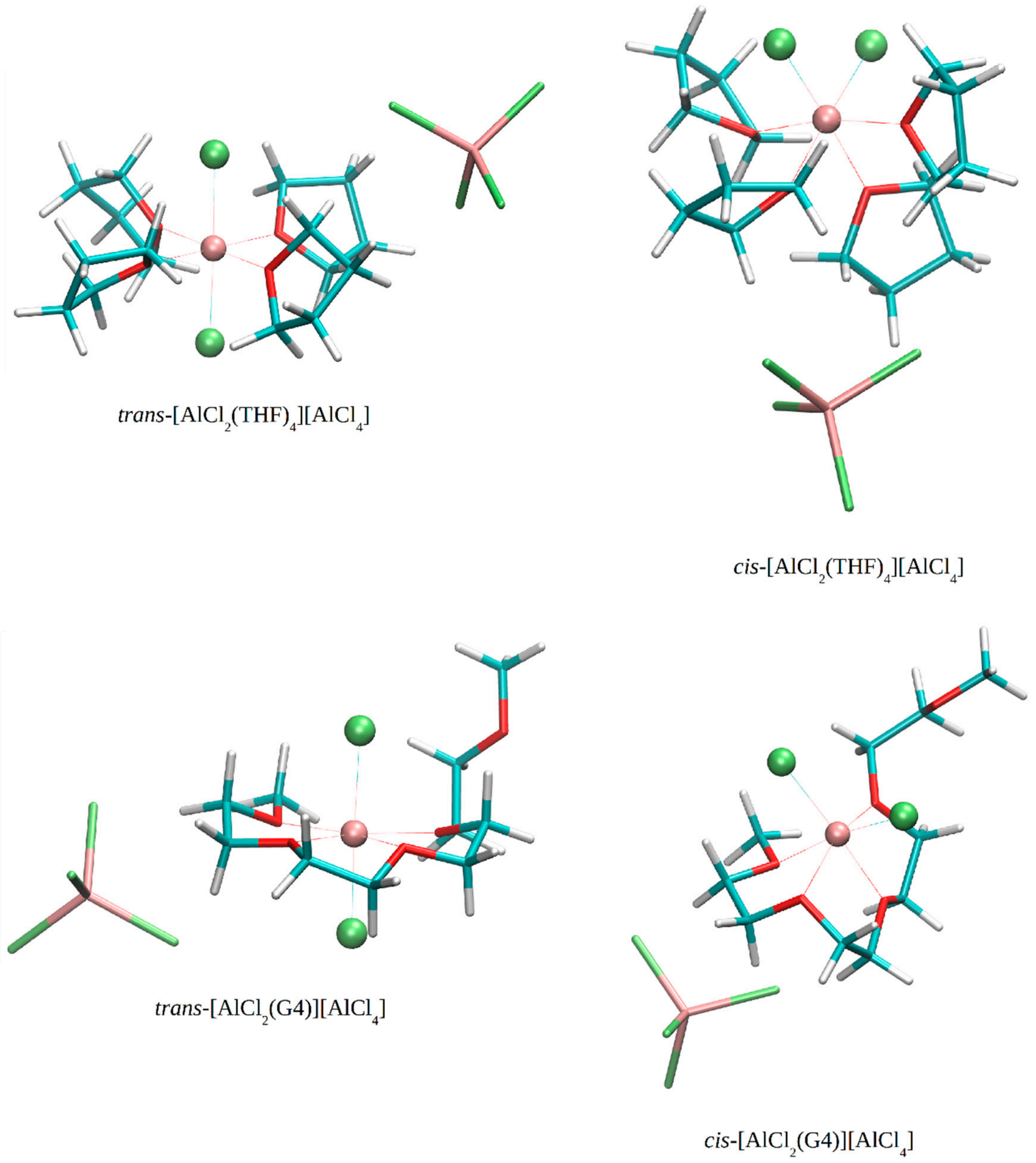

Figure S4. Structures of neutral [AlCl$_2$]$^+$ solvates in THF (top panels) and in G4 (bottom panels) obtained from the QC calculations at the ωB97XD/aug-cc-pVDZ level.

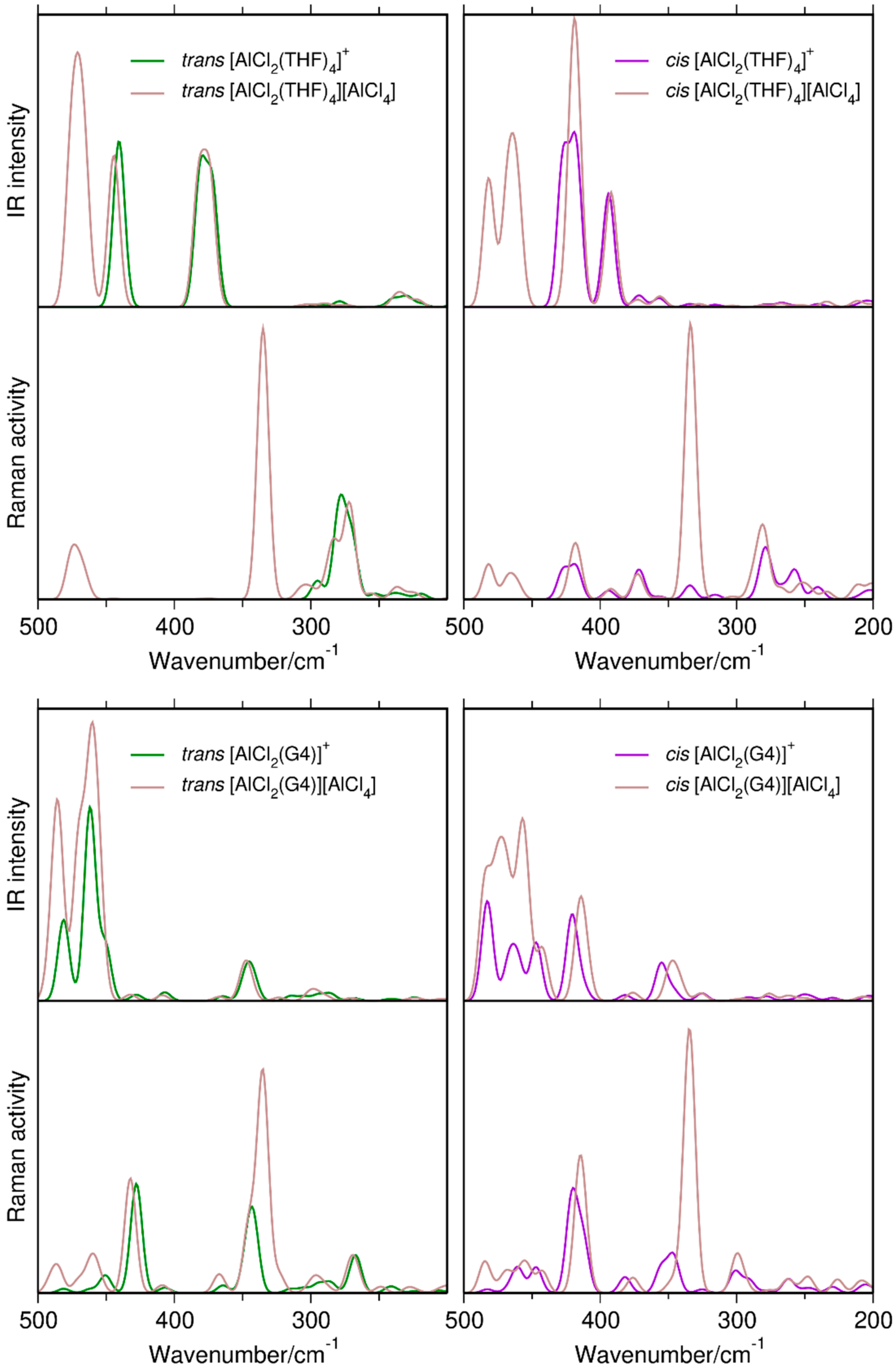

Figure S5. IR and Raman spectra of charged and neutral $[AlCl_2]^+$ solvates in THF (top panels) and in G4 (bottom panels) obtained from the QC calculations at the ωB97XD/aug-cc-pVDZ level.

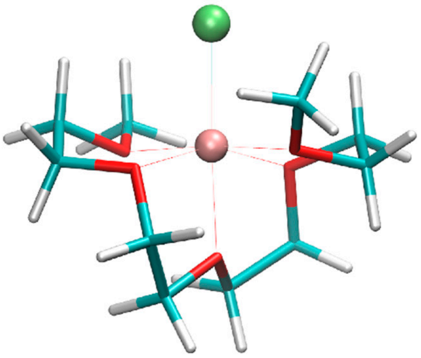

$[AlCl(G4)]^{2+}$

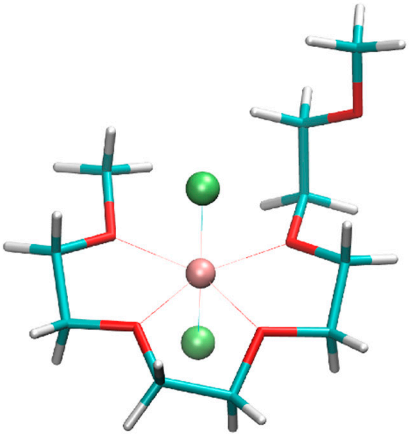

trans-$[AlCl_2(G4)]^{+}$

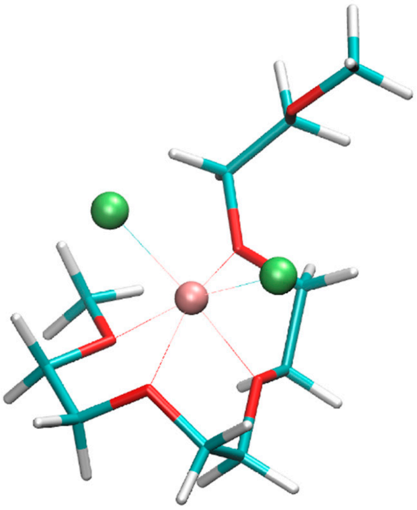

cis-$[AlCl_2(G4)]^{+}$

Figure S6. Structures of $AlCl_n$ solvates in G4 obtained from the QC calculations at the ωB97XD/aug-cc-pVDZ level.
